# Wire internal fixation: an obsolete, yet valuable method for surgical management of facial fractures

**DOI:** 10.11604/pamj.2014.17.219.3398

**Published:** 2014-03-19

**Authors:** Rasmané Béogo, Pierre Bouletreau, Tarcissus Konsem, Ibraïma Traoré, Antoine Toua Coulibaly, Dieudonné Ouédraogo

**Affiliations:** 1 Department of Oral and Maxillofacial Surgery, CHU Sanou Souro, Burkina Faso; 2 Department of Stomatology and Maxillofacial Surgery, CHU Lyon Sud, France; 3 Department of Oral and Maxillofacial Surgery, CHU Yalgado Ouédraogo, Burkina Faso

**Keywords:** Facial fracture, wire internal fixation, outcome

## Abstract

In some developing countries wire is still widely used in facial fractures internal fixation. This study presents the effectiveness and complications of wire osteosynthesis in a university teaching hospital in Burkina Faso and discusses some of its other benefits and disadvantages. Notes of 227 patients with facial fractures treated by wire internal fixation at department of stomatology and maxillofacial surgery of CHU Souro Sanou, Burkina Faso between 2006 and 2010 are reviewed retrospectively. A satisfactory treatment outcome was recorded in 91.2% of the 227 patients. Complications occurred in 8.8% of the patients who had operative site infection (3.1%), malocclusion (1.8%), sensory disturbance (1.8), facial asymmetry (1.3%), delayed bone union (0.9%) or enophtalmos (0.4%). The overall complications rate was 7.4% after mandibular osteosynthesis, 6.9% after Le Fort osteosynthesis and 6.5% after zygoma osteosynthesis. Post operative infections occurred irrespective to the surgical site. The other complications were more specific to the surgical site. Wire internal fixation may be a reasonable alternative for the surgical treatment of non-comminuted facial fractures and those without bone substance loss, in the setting of limited resources.

## Introduction

Facial fractures may indicate accurate reduction and stable fixation because of their displacements and resulting functional and morphological consequences. This is at best achieved by open reduction and internal fixation [1]. Plates and screws technology is the gold standard in the modern treatment and widely used in developed countries whilst wire fixation fell in abandonment. However, in the third world where facial fractures comprise a significant proportion of trauma, lack of resources precludes the use of plating technology in most of countries and wire osteosynthesis is still widely used [2, 3]. However, there are few data published on the results of wire osteosynthesis from developing countries. This study aims to evaluate the effectiveness and complications of this traditional method of treatment in a university teaching hospital in Burkina Faso and to discuss some of its other benefits and disadvantages.

## Methods

This is a retrospective descriptive study carried out between 2006 and 2010 at the department of stomatology and maxillofacial surgery of CHU Souro Sanou, a university teaching hospital in Burkina Faso. All patients seen during the period who underwent facial bone fracture treatment by wire osteosynthesis were included. A total number of 227 patients were included. Their mean age was 31.9 years (SD 12.3) and 63.1% of them had between 20 and 39 years. The aetiologies of the fractures were road traffic crashes (80.6%), interpersonal violence (10.5%), falls from heights (5.3%), sport related accidents (2.3%) and occupational accidents (1.3%). Osteosynthesis was performed in 137 zygomatic fracture patients, 121 patients with mandibular fracture, and 43 with Le Fort fracture. In 30.8% (70/227) of the patients, two or three bones were involved.

Indications of open reduction and wire fixation were: displaced zygoma fractures resulting in mouth opening restriction, diplopia, enophtalmous or face asymmetry; displaced Le Fort or mandible fractures with occlusion impairment or poor dentition. All the patients had radiographic evidence of displaced fractures.

The surgery was performed according to the theatre schedule, usually within 6 to 10 days after the fracture occurrence. The patient was subjected to assessment of the general condition and general anaesthesia. Intraoral, external, or combined approaches were used. A 0.5mm-diameter soft stainless steel wire was used for the fixation. At each fracture focus of the mandible body or angle, two points of fixation were performed, on the base of mandible and the oblique line. In Le Fort II fractures, osteosynthesis was performed on the nasal cavity lateral rim and the maxillozygomatic buttress. In Le Fort III fractures, it was performed on the infra orbital rim and on the frontozygomatic suture. In the zygoma fractures, a 3-point fixation across zygomaticomaxillary buttress, frontozygomatic suture, and inferior orbital rim or a 2-point fixation across zygomaticomaxillary buttress and frontozygomatic buttress were performed after evaluation of reduction of the fracture at these three regions; temporozygomatic suture displaced fractures were subjected to closed reduction.

The transosseous wiring was always combined with the jaws immobilization in mandibular or Le fort fracture patients. This immobilization was recommended for six weeks in adult patients and three weeks in children. It consisted in maxillomandibular fixation (MMF) using arch bars in patients with mandibular fractures and fixation with wires from the zygomatic process of the frontal bone to the lower arch bar in patients with maxillary fractures. Antibiotics were given preoperatively and continued for 7 to 10 days after surgery. A steroid anti inflammatory was given for 3 days preoperatively and continued for 3 to 4 days postoperatively. Chlorhexidine mouth rinse was given when intraoral approach was used. Liquid and soft diet was recommended in patients with jaws immobilization.

Patients were followed-up postoperatively for at least, 3 weeks in the zygoma osteosynthesis and 6 weeks in the mandible and Le Fort osteosynthesis. At follow-up, patients with mandibular or Le Fort fractures were assessed for occlusion. Those with zygoma fractures were checked for diplopia, enophtalmos and face asymmetry. All the patients were checked for face nervous impairment, operative site infection, and bone union impairment. Operative site infection was defined by a painful swelling or abscess formation with or without drainage from the fracture site. Delayed bone union referred to persistent mobility at the fracture site 8 weeks after osteosynthesis [4].

## Results

Of the 227 patients, 207 (91.2%) had an uneventful and satisfactory treatment outcome as defined by a timely bone healing, restoration of the face morphology and the functions. Twenty patients (8.8%) had 21 complications (1 patient had 2 complications). Operative site infection was the most common complication (Table 1). It consisted in surgical wound infection which resolved after systemic and local anti infectious treatment. Sensory disturbance consisting in hypoesthesia or anaesthesia of the cheek, the lip, the chin, ipsilateral to the surgical side recovered in all the patients within 3 to 5 months postoperatively. Delayed bone union occurred in two patients who presented respectively a comminution and a mild substance loss of the mandible. One patient presented enophtalmos after orbital floor substance loss repaired with autologous bone graft.

**Table 1:** Complications in 227 facial fracture patients following wire osteosynthesis

Complications	Number of cases	% (n = 227)
Operative site infection	7	3.1
Malocclusion	4	1.8
Sensory disturbance	4	1.8
Facial asymmetry	3	1.3
Delayed bone union	2	0.9
Enophtalmos	1	0.4
**Total**	21	8.8

The overall complications rate was 7.4% after mandibular osteosynthesis, 6.9% after Le Fort osteosynthesis and 6.5% after zygoma osteosynthesis. The difference was not statistically significant (p = 0.2). Post operative infections occurred irrespective to the surgical site. The other complications were more specific to the surgical site

## Discussion

Open reduction and internal fixation of facial fracture has the multiple challenges of restoring the face anatomy and functions impaired by the displacement of the fracture segments and avoiding the treatment-related complications. In this study, these goals are achieved in more than 90% of the patients who had a satisfactory outcome after wire osteosynthesis. Internal wire fixation ensures alignment and contact of the fracture fragments. In a Le Fort or a mandible fracture, these actions are secured by the jaws immobilization. In the zygomatic complex fracture management Gandi et al. declare wire as efficient as miniplate [5].

Transosseous wiring osteosynthesis is an economical method of internal fixation. Wire and arch bars are more easily affordable than plates and screws. Unlike in the plate and screw system, there is no need of calibrated drill. Moreover, wire is a well tolerated and non-cumbersome material which does not require removal. In this study, no patient complained of wire intolerance as frequently encountered with plates. Erol et al. report complaint of “cold feeling” in cold weather in patients after zygomatico-orbital fracture treatment by miniplate osteosynthesis [6]. Chakranarayan et al. report plates removal in two patients warranted by complaint of palpable implant at the frontozygomatic region and implant rejection [7]. Some of wire internal fixation disadvantages include the facts that it is not strong enough to prevent interfragmentary motion across the fracture [8] and lack of directional control. Jaws fixation required for stability enforcement in the mandibular or Le Fort fracture treatment may result in a significant weight loss due difficulties of feeding, adverse effects on the patient’s social and professional life due to speech difficulty [6]. Additionaly, difficulties in maintaining dental hygiene result lastly in dental and periodontal diseases and development of pulmonary atelectasis is reported [9]. Lack of stability may result in bone non-union as any mobility of the fracture fragments impedes the bony healing. Challenge of passing the wire through the drill holes especially in case of limited exposure of the fracture focus and the wire break during its tightening are other concerns which make wire internal fixation time consuming. Additionally, there is a risk of iatrogenic bone substance loss when passing the wire or during its tightening, particularly on the upper jaw bones or in case of comminuted fracture.

Postoperative complications of facial fracture are broad and include occlusion, mouth opening, vision, face sensory and bone union impairments, infections, and face asymmetry. Post operative infections, the most common complication in this study are a usual concern in facial fracture surgery, irrespective to the facial bone [10, 11]. Their spectrum varies from the surgical wound infection to osteomyelitis. Their rates in literature are diverse and the variability may be due to lack of clear definition of post operative infection [11], differences of study designs and reporting bias. Although the operative site infection rates according to the fracture location do not reach significant differences in this study, mandible fractures are reported to be the most common provider of infectious complications [12]. Postoperative extended regimen prophylactic antibiotics as routinely performed in this study is reported to have no significant beneficial effect [13, 14]. Several studies report compound fracture and delay in treatment being the most determinant risk factors [13, 15, 16]. Maloclusion may result from inadequate reduction and fixation of a Le Fort or a mandible fracture. However, a restored occlusion may be compromised by a non-compliant patient releasing prematurely himself the jaws immobilization. Delayed bone union observed in two patients with mandible fracture in this study can be due to lack of stability of the fixation as loss of substance or communition of the fracture focus can not be bridged effectively by wire. Face asymmetry and enophtalmos after zygomatic complex fracture repair are likely due to improper treatment. The zygoma provides the prominence of the cheek by its convex external surface and forms a part of the bony orbit. Its fractures are commonly quadripod, at the four processes attached to the frontal, maxillary, temporal and sphenoid bones [17–18] resulting to the bone displacement inferiorly, medially and rotation. Any inaccurate repositioning of the bone at its different wrists may result in the cheek depression or flattening as well as enophtalmos. In all the patients in this study, fixation of zygomatic complex at more than one point is performed as recommended to achieve a definite stability [7]. However, initial proper reduction in some patients may have been compromised postoperatively by masticatory forces as a rigid and stable fixation could be hardly achieved with wire osteosynthesis. Enophtalmos is a surgical challenge with a fracture of the inferior wall or the medial wall of the orbit reported to be the most common factors [19, 20]. Enophthalmos in the patient in this study could be due the defect of the inferior orbital as well as an outward displacement of the zygomatic bone and loss of substance of the greater wing of the sphenoid bone at the lateral wall of the orbit as reported by some authors [21, 22]. Such treatment of enophtalmos could be hardly achieved as the patient did not have preoperative computed tomography.

## Conclusion

Since the plating technology is not easily affordable in developing countries, wire internal fixation may be a reasonable alternative for the surgical treatment of non-comminuted facial fractures and those without bone substance loss, in such setting. However, efforts should be directed to adopt the modern technology of plating system because of its better results.
